# Structural evolution of the selectivity clamp confers ADPR-PP specificity in Namat, a phage nicotinamide ADP-ribose transferase

**DOI:** 10.1093/nar/gkaf1492

**Published:** 2026-01-06

**Authors:** Meimei Lan, Li Xu, Yizhen Han, Tong Cui, Zhi Qiao, Yan-Bin Teng, Na Wang, Hongyu Bao

**Affiliations:** Department of Biochemistry and Molecular Biology, School of Life Sciences, Anhui Medical University, Hefei, Anhui 230032, China; Institute of Bio-Architecture and Bio-Interactions, Shenzhen Medical Academy of Research and Translation, Shenzhen 518107, Guangdong Province, China; Department of Biochemistry and Molecular Biology, School of Life Sciences, Anhui Medical University, Hefei, Anhui 230032, China; Department of Biochemistry and Molecular Biology, School of Life Sciences, Anhui Medical University, Hefei, Anhui 230032, China; Department of Biochemistry and Molecular Biology, School of Life Sciences, Anhui Medical University, Hefei, Anhui 230032, China; Department of Biochemistry and Molecular Biology, School of Life Sciences, Anhui Medical University, Hefei, Anhui 230032, China; Department of Bioengineering, School of Life Sciences and Medical Engineering, Anhui University, Hefei 230601, China; Department of Biochemistry and Molecular Biology, School of Life Sciences, Anhui Medical University, Hefei, Anhui 230032, China

## Abstract

Phages and bacteria engage in an evolutionary arms race, in which NAD⁺ depletion serves as a potent bacterial defense. The phage NAD⁺ reconstitution pathway 1 (NARP1) counteracts this strategy via ADP-ribose phosphorylase (Adps) and nicotinamide ADP-ribose transferase (Namat), which restores NAD⁺ by repurposing the products of NAD⁺-depletion systems. Here, we dissect how Namat, the key ligase of NARP1, combines a conserved Nampt-like catalytic core with a specialized adenine ring-binding selectivity clamp to overcome host immunity. We determine its crystal structures bound to nicotinamide (NAM) and NAD⁺, combined with mutational, biochemical, and phylogenetic analyses. The structures reveal a “selectivity clamp,” consisting of a variable loop and a conserved helix, that enforces strict specificity for ADP-ribose pyrophosphate (ADPR-PP) over phosphoribosylpyrophosphate (PRPP), the substrate of nicotinamide phosphoribosyltransferase (Nampt). Functional assays show that both the catalytic center and the selectivity clamp are essential for NAD⁺ biosynthesis and for counteracting NAD⁺-depleting defense. Guided by these insights, we identify bacterial homologs of NARP1 with similar enzymatic activity. These findings define the structural basis of Namat substrate selectivity and refine our understanding of NAD⁺ metabolism in host–phage interactions.

## Graphical Abstract

**Graphical Abstract:**
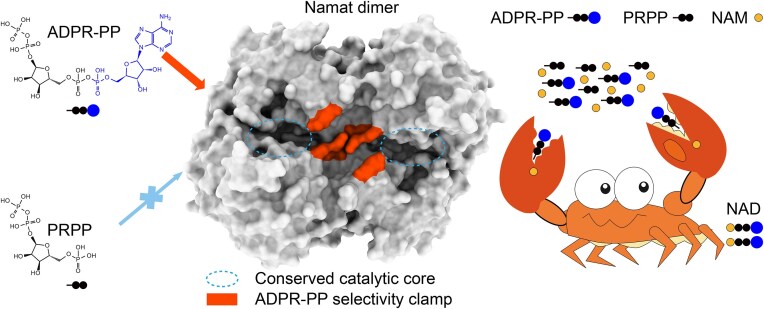

## Introduction

The widespread use of small-molecule antibiotics in medicine and agriculture has imposed relentless selective pressure on bacteria, driving the emergence of multidrug resistance and creating a global public health crisis [1]. Bacteriophages (phages), viruses that specifically infect and kill bacteria, have gained renewed attention as promising alternatives to antibiotics due to their host specificity and lower likelihood of causing adverse effects [2–4].

For over three billion years, bacteria and phages have co-evolved in a continuous molecular arms race [5]. Bacteria deploy multilayered defense systems to block phage invasion and replication [6–13], whereas phages develop countermeasures that disrupt host immunity [8, 14–19]. Most of these countermeasures act as precision inhibitors, targeting specific immune modules or their signaling molecules to suppress bacterial defenses [14, 16]. To date, researchers have identified >150 distinct bacterial defense systems and over 140 phage-encoded anti-defense systems. Investigating this coevolutionary interplay provides critical insights with broad implications for medicine, agriculture, and food safety [2, 4, 20].

NAD⁺ is an essential cofactor for redox balance and energy metabolism [21]. Beyond redox reactions, NAD⁺ is a non–oxidative substrate for ADP–ribosyltransferases that modify proteins, DNA, and RNA with ADP–ribose (ADPR), regulating DNA repair, chromatin structure, transcription, and antiviral responses [22–25], and thus tightly coupling NAD⁺/ADPR homeostasis to nucleic acid metabolism. Upon detecting phage infection, many bacterial defense systems deplete cellular NAD⁺ [25, 26]. Genomic surveys reveal that this strategy is widespread: over 7% of sequenced bacterial genomes encode NAD⁺-depletion modules, including Kongming [27], Thoeris [28], Pycsar, CBASS, AVAST [11], DSR, SIR2–HerA [29], pAgos (SPARTA and SPARSA) [30], SEFIR [10], and Retron-Eco1 [31]. These systems employ TIR [32], SIR2 [29], or SEFIR [10] domains that, once activated, cleave NAD⁺ into nicotinamide (NAM) and ADPR, thereby triggering dormancy or cell death to block phage replication. Consequently, phages must evolve countermeasures to restore NAD⁺ balance.

**Figure 1.**
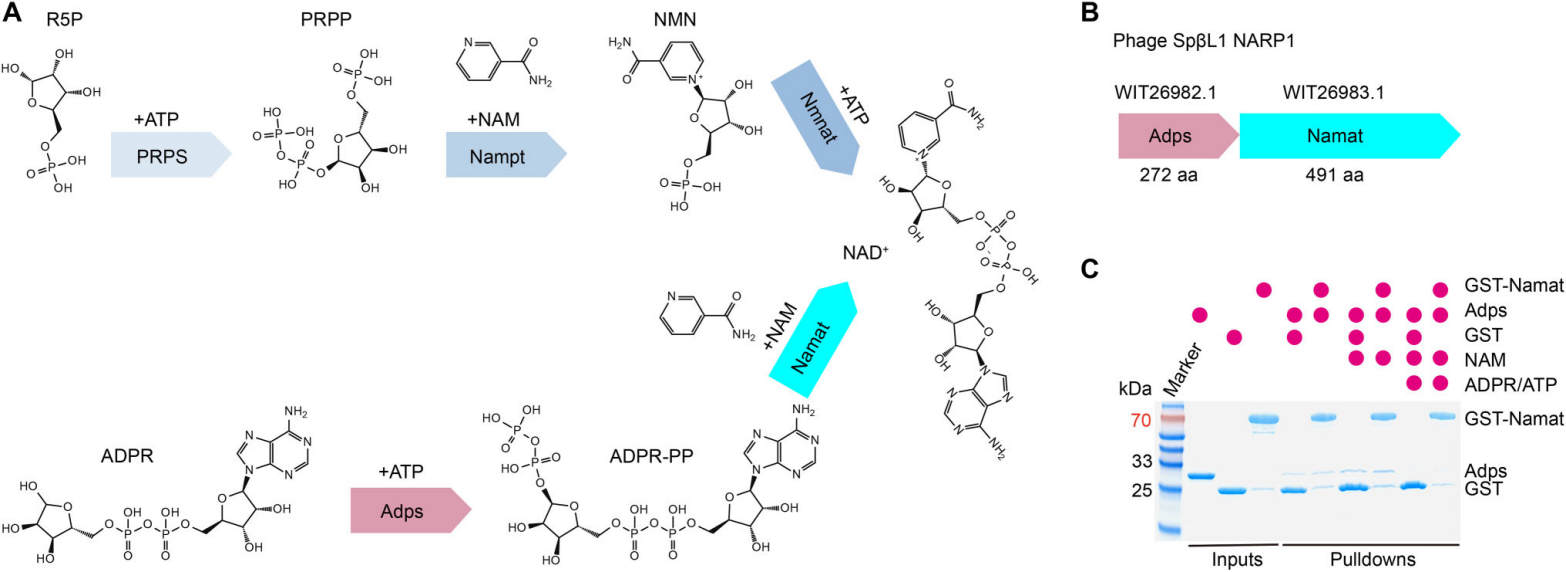
Overview of NARP1.(**A**) Enzymatic reactions of the canonical NAD^+^ salvage pathway (top) and NARP1 (bottom). (**B**) Genome organization of the SpβL1 NARP1. (**C**) GST pull-down assay demonstrating the absence of a stable Namat–Adps complex, regardless of substrate availability (NAM, ADPR, or ATP).

The phage-encoded NAD⁺ reconstitution pathway 1 (NARP1) is a recently discovered phage anti-defense system that repurposes ADP-ribose (ADPR) and nicotinamide (NAM), the products of bacterial NAD⁺-depletion defenses, to regenerate NAD⁺ [33]. In contrast to the precision inhibitors described earlier, NARP1 reestablishes cellular energy homeostasis, enabling phages to counteract various NAD⁺-depleting systems. The pathway comprises two enzymes, Adps and Namat, which functionally parallel PRPS and Nampt of the canonical salvage pathway, but act on distinct substrates (Fig. 1A). Specifically, Adps phosphorylates ADPR to generate ADPR-PP, analogous to PRPS converting ribose-5-phosphate into PRPP [34], whereas Namat ligates ADPR-PP with NAM to generate NAD⁺, analogous to Nampt condensing PRPP with NAM to produce nicotinamide mononucleotide (NMN) [35]. In contrast, the canonical salvage pathway requires an additional step, in which NMN adenylyltransferase (Nmnat) adenylylates NMN to form NAD⁺ [36]. Thus, NARP1 regenerates NAD⁺ in only two steps, providing a streamlined alternative to bypass bacterial NAD⁺ depletion. However, the substrate selectivity mechanisms of both Adps and Namat remain obscure.

In this study, we elucidate the molecular basis of Namat substrate selectivity within the NARP1 pathway. We determined the crystal structures of Namat from the SpβL1 phage in complex with NAM and NAD⁺, together with mutational, functional, and phylogenetic analyses. These studies reveal that Namat couples a conserved Nampt-like catalytic core with an adenine ring-binding selectivity clamp that confers specificity for ADPR–PP over PRPP. The clamp is formed by a variable selectivity-clamp loop (SCL) and a conserved selectivity-clamp helix (SCH). Comparative analyses reveal that the SCL provides a nonconserved residue contributing to ADPR-PP’s adenine recognition, whereas the SCH has evolved additional aromatic residues that stabilize adenine binding. Mutagenesis demonstrates that both the catalytic center and the clamp are indispensable for enzymatic activity, and for counteracting bacterial NAD⁺-depleting defenses. Furthermore, phylogenetic and structural comparisons identify NARP1 homologs in certain bacteria, which retain NAD⁺ biosynthetic activity *in vitro* and *in vivo*. These observations indicate that NARP1 is not restricted to phages and broaden current perspectives on the evolutionary diversification of NAD⁺ metabolism.

## Materials and methods

### Plasmid construction

All coding sequences, including SpβL1 Adps, SpβL1 Namat, Paenibacillus foliorum Adps (pfAdps), pfNamat, R1DNK, and the KomABC operon with their native promoters, were synthesized by GenScript (Nanjing, China). For simplicity, SpβL1 Adps and SpβL1 Namat are hereafter referred to as Adps and Namat, respectively. For recombinant protein expression, the Adps and pfAdps genes were cloned into the pET22b vector (NdeI–XhoI), the Namat gene into the pGEX-6P1 vector (BamHI–SalI), and the pfNamat gene into the multiple cloning sites MCS1 of pCDFDuet-1 (BamHI–SalI). Human Nampt (pT7-Nampt) was purchased from the Miaoling Plasmid platform (Wuhan, China). Point mutations were introduced by standard polymerase chain reaction mutagenesis and confirmed by Sanger sequencing.

For *Escherichia coli* cell viability assays, R1DNK and the KomABC operon were cloned as described previously [27]. Briefly, the R1DNK gene was inserted into the pBAD/His_A vector to allow induction with L-arabinose and repression with glucose. The KomABC operon, including its native promoter, was cloned into the pCDFDuet-1 vector; the T7 promoter and lacI cassette were removed to achieve constitutive expression. Finally, the Adps gene (under its native promoter) and Namat (wild type or mutant) were cloned into the multiple cloning sites MCS1 and MCS2 of pRSFDuet-1, respectively, using the ClonExpress II One Step Cloning Kit (Vazyme, Nanjing). As with the KomABC construct, the T7 promoter and lacI cassette were removed, enabling constitutive co-expression of Adps and Namat in *E. coli*. For pfNARP1, pfAdps was cloned into MSC2 of pCDFDuet-pfNamat (NdeI–XhoI). The pRSFDuet-SPARSA plasmid was generously provided by Dr Ning Jia (School of Medicine, Southern University of Science and Technology, Shenzhen, China).

### Protein expression and purification

For protein production, *E. coli* BL21(DE3) cells were transformed with most of the respective plasmids and cultured in Luria–Bertani (LB) medium supplemented with appropriate antibiotics at 37°C until OD600 reached 0.8–1.0. Protein expression was induced with 0.4 mM isopropyl β-D-1-thiogalactopyranoside (IPTG) and allowed to proceed overnight at 20°C. For production of pfAdps and pfNamat, *E. coli* BL21(AI) cells were transformed with pET22b-pfAdps or pCDFDuet-pfNamat, respectively, and cultured under the same conditions, except that expression was induced with both 0.4 mM IPTG and 0.2% L-arabinose.

For purification of His-tagged Adps, pfAdps, pfNamat, and human Nampt, cells were resuspended in buffer A (20 mM Tris–HCl, pH 7.5, 0.5 M NaCl, 1 mM phenylmethylsulfonyl fluoride (PMSF) ), lysed by sonication, and centrifuged at 15 000 × *g* for 1 h at 4°C. The supernatant was incubated with High-Affinity Ni-NTA resin (GenScript) for 1 h at 4°C, washed with buffer B (20 mM Tris–HCl, pH 7.5, 0.5 M NaCl, 25 mM imidazole), and eluted with buffer C (20 mM Tris–HCl, pH 7.5, 0.2 M NaCl, 500 mM imidazole). Eluted proteins were concentrated and further purified on a HiLoad 16/600 Superdex 200 column (Cytiva) equilibrated with buffer C supplemented with 2 mM dithiothreitol (DTT).

For purification of GST–Namat, cells were resuspended in buffer D (20 mM Tris–HCl, pH 7.5, 0.5 M NaCl, 2 mM DTT, 1 mM PMSF), lysed by sonication, and centrifuged as above. The supernatant was incubated with GST Chromrose 4FF resin (Qingdao CHROMSEP, China) for 1 h at 4°C. The resin was then washed with buffer E (20 mM Tris–HCl, pH 7.5, 0.5 M NaCl), and GST–Namat was eluted with buffer F (25 mM Tris–HCl, pH 7.5, 1 M NaCl, 20 mM reduced glutathione). The GST–Namat was further purified by size-exclusion chromatography on a Superdex 200 column equilibrated in buffer C. For untagged Namat, the GST tag was cleaved with in-house 3C protease before gel filtration.

The molar mass of Namat was determined by Refeyn mass photometry [37], 50 nM of Namat after gel filtration was used.

### GST pull-down assays

To test for an interaction between Namat and Adps, 1 nmol of GST–Namat and 1 nmol of Adps were mixed in 100 µl of buffer G (20 mM Tris–HCl, pH 8.0, 150 mM NaCl, 2 mM MgCl₂, 2 mM DTT), with or without NAM, ADPR, and ATP, and incubated for 1 h at 30°C. Then, 20 µl of GST Chromrose 4FF resin was added, and the mixture was incubated for an additional hour at 4°C. The beads were washed four times with buffer H (20 mM Tris–HCl, pH 8.0, 150 mM NaCl, 2 mM MgCl₂, 2 mM DTT, 0.5% Triton X-100), resuspended in sodium dodecyl sulfate–polyacrylamide gel electrophoresis (SDS–PAGE) loading buffer, and analyzed by 12.5% SDS–PAGE.

### Crystallization, X-ray data collection, and structure determination

Crystals of Namat were obtained by sitting-drop vapor diffusion at 18°C. Namat (6 mg/ml) pre-incubated with 2 mM NAM was crystallized in 1.5 M ammonium sulfate and 0.1 M Bis-Tris propane (pH 7.0). Namat (6 mg/ml) pre-incubated with 2 mM NAD⁺ was crystallized in 0.1 M NaCl, 0.1 M HEPES (pH 7.5), and 1.6 M ammonium sulfate. Crystals were cryoprotected with mother liquor supplemented with 25% glycerol and flash-frozen in liquid nitrogen. X-ray diffraction data were collected at the SSRF beamlines BL18U1 and BL19U1 [38–40], and the data were processed with XDS [41], AutoPX [42], and CCP4 [43].

The Namat–NAD⁺ structure was solved by molecular replacement in PHASER [8] using the human Nampt structure (PDB ID 6E68) as a search model. Models were manually built in Coot [44] and refined in PHENIX [45]. The Namat–NAM structure was then solved using the refined Namat–NAD⁺ model as a search template. Structural figures in this study were prepared with PyMOL and UCSF ChimeraX [46].

### *In vitro* NAD⁺ generation assay

Reactions (50 mM Tris–HCl, pH 7.5, 12 mM MgCl₂, 0.02% Bovine serum albumin (BSA), 0.5 mM ATP, 0.1 mM ADPR, 0.5 µM Adps) were incubated at 30°C for 30 min to generate ADPR-PP. Subsequently, 0.1 mM NAM and 0.2 µM Namat (or mutant enzymes) were added, and the reactions were continued for another 30 min. NAD⁺ was quantified using a commercial NAD⁺/NADH assay kit (Beyotime S0175) by measuring absorbance at 450 nm. All assays were performed in triplicate. NAD⁺ generation assays with pfAdps and pfNamat were conducted under the same conditions. Human Nampt was assayed in parallel at both 30 and 37°C.

### *In vitro* NMN generation assay

Reactions were performed by incubating 0.2 μM of the respective enzyme (Namat, its mutants, or Nampt as a control) with 0.1 mM NAM and 0.1 mM PRPP in a reaction buffer containing 50 mM Tris–HCl (pH 7.5), 0.02% BSA, and 12 mM MgCl_2_. The enzymatic reactions were carried out for 1 h at 37°C, and the NMN was quantified by a fluorescence-based assay [47, 48]. Briefly, 25 μl reaction mixture or an NMN standard was mixed with 10 μl of 20% acetophenone (in DMSO) and 10 μl of 2 M KOH, incubated on ice for 10 min, then supplemented with 45 μl of 100% formic acid and incubated at 37°C for 15 min. Finally, 40 μl of each sample was transferred to a 384-well plate, and fluorescence was measured on a TECAN SPARK plate reader at 355 nm excitation and 435 nm emission.

### *In vivo E. coli* cell viability assays

To evaluate anti-defense activity, *E. coli* MG1655 cells were co-transformed with pRSFDuet-Adps-Namat (or mutant versions), pBAD-R1DNK, and pCDFDuet-KomABC. Transformed cells were grown overnight in MMB medium (LB supplemented with 0.1 mM MnCl_2_ and 5 mM MgCl_2_) containing kanamycin, streptomycin, and ampicillin (50 µg/ml each) at 37°C. Overnight cultures were diluted 1:100 and grown to an OD_600_ of 0.6–0.8, harvested by centrifugation, washed, and resuspended in PBS. Tenfold serial dilutions (10^−1^ to 10^−6^) were prepared, and 5 µl of each dilution was spotted onto MMB plates supplemented with antibiotics and either 0.4% L-arabinose or 2% glucose.

The *in vivo* NAD^+^-generation activity of pfNARP1 was assessed using the SPARSA system [49]. *Escherichia coli* BL21(AI) cells were co-transformed with pRSFDuet-SPARSA and either pCDFDuet-1 or pCDFDuet-pfNARP1. Transformed cells were grown overnight in LB medium containing kanamycin and streptomycin (50 µg/ml each) at 37°C. Cultures were diluted 1:100 and grown to an OD_600_ of 0.6–0.8, then collected by centrifugation, washed, and resuspended in PBS. The cells were serially diluted (10^−1^ to 10^−6^), and 5 µl of each dilution was spotted onto LB plates supplemented with antibiotics and inducer (0.4%L-Arabinose and 0.25 mM IPTG) or 2% glucose.

### Phylogenetic analyses of Namat and Nampt

Sequence of Namat and Nampt homologs in phages were obtained from a previous research [33], which screened for in 20 185 phage genomes from the INPHARED database. Bacterial Namat homologs were identified using the SpβL1 Namat full-length sequence (UniProt ID: A0A9Y1YVZ0) as a query and were retained only if an adps gene was located within 10 genes in the genomic vicinity. Bacterial and eukaryotic Nampt homologs were retrieved from UniProt using full-length sequences from *Xanthomonas campestris* (UniProt ID: A0A0H2 × 5R2) and Homo sapiens (UniProt ID: P43490), respectively. To simplify the analysis, representative Namat and Nampt sequences from diverse species were selected. In total, 135 sequences were aligned using Clustal Omega [50]. Phylogenetic trees were constructed with MEGA12 with the maximum likelihood method [51]. Sequence conservation was visualized as sequence logos generated with WebLogo [52].

### Manuscript preparation

ChatGPT (OpenAI, San Francisco, CA, USA) was employed to improve the grammar and readability of the manuscript text. All intellectual contributions, data analyses, and interpretations were made by the authors.

## Results

### Namat does not form a stable complex with Adps

The SpβL1 phage encodes Adps and Namat within a single operon (Fig. 1B). In prokaryotes, proteins co-encoded in operons frequently function as complexes, suggesting that Adps and Namat may physically interact. To test whether Adps and Namat function as a physical complex, we performed GST pull-down assays using purified proteins. After incubation under physiological conditions, the mixtures were applied to GST affinity resin and analyzed by SDS–PAGE. No co-elution of Adps with Namat was detected under all tested conditions, including in the presence or absence of NAM, ADPR, and ATP (Fig. 1C), indicating that the two proteins do not form a stable complex *in vitro*, although transient interactions cannot be ruled out. These results suggest that Adps and Namat act sequentially within the NARP1 pathway, with Adps generating the phosphorylated intermediate ADPR-PP, which is subsequently ligated with NAM by Namat to regenerate NAD⁺.

### Crystal structure of Namat bound to NAM

**Figure 2.**
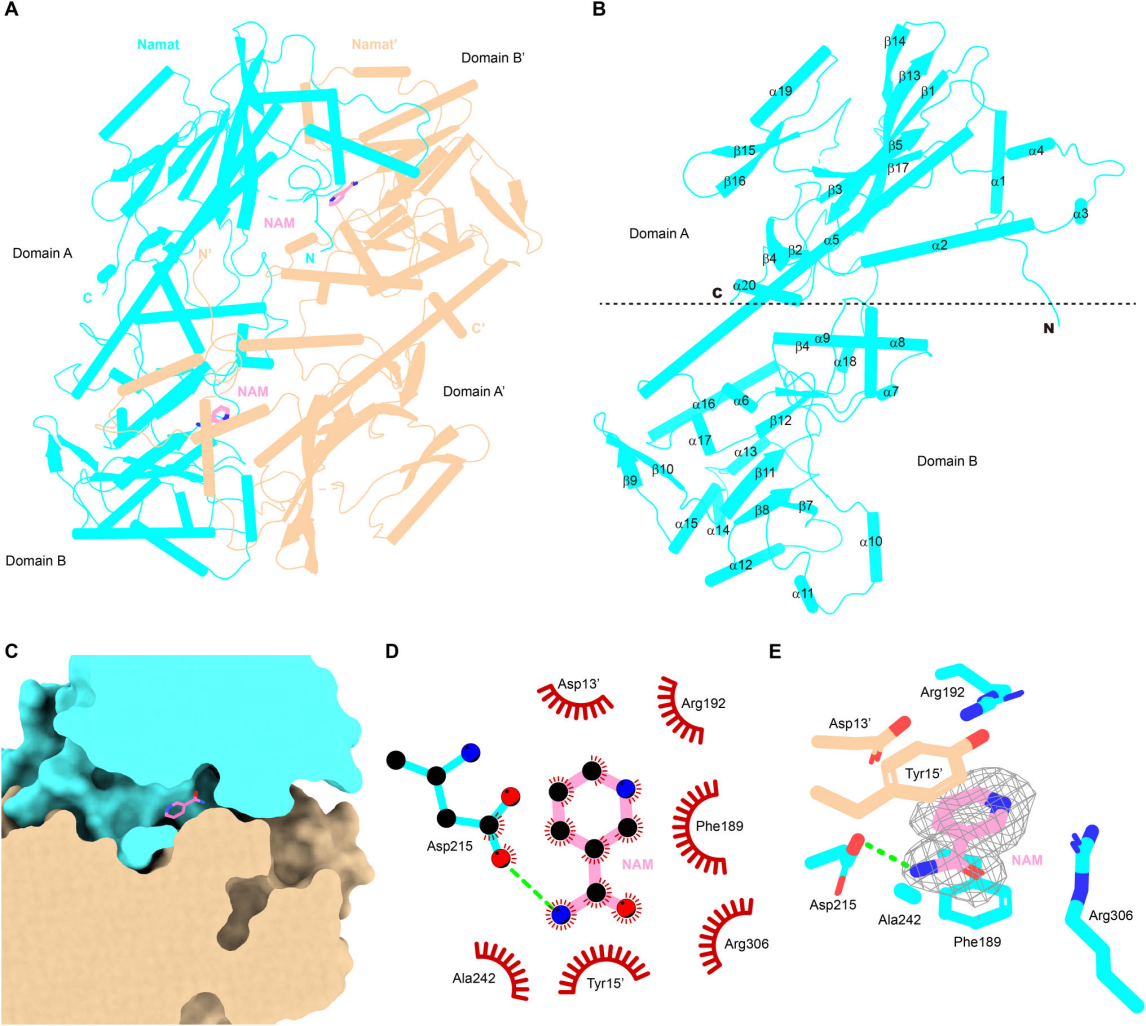
Crystal structure of SpβL1 Namat in complex with NAM. (**A**) Ribbon diagram of Namat dimer in complex with NAM. One protomer is colored in cyan, and the other in wheat. (**B**) Ribbon diagram of a single Namat protomer. (**C**) Slice view of the NAM-binding pocket. 2D LigPlot representation (**D**) and stereo ribbon diagram (**E**) of key interactions between Namat residues and NAM. The 2mFo-DFc electron density map (contoured at 1.0 sigma) of NAM is shown as gray mesh. Residues involved in hydrophobic interactions are shown as spoked arcs. Hydrogen bonds are indicated by green dashed lines.

To elucidate the molecular basis of NAM recognition, we determined the crystal structure of Namat in complex with NAM at 2.4 Å resolution (Supplementary Table S1). Namat is a physical and structural dimer (Fig. 2A and Supplementary Fig. S1), with each protomer (Namat and Namat’) comprising 20 α-helices and 17 β-strands (Fig. 2B). The two protomers are structurally identical, with an overall Cα root mean square deviation (RMSD) of 0.189 Å (Supplementary Fig. S2). The loop between β14 and β15 is disordered in both protomers. Each protomer can be divided into two domains (Fig. 2B), A and B. Domain A contains seven α-helices (α1–α4, the N-terminus of α5, α19, and α20) and 10 β-strands (β1–β5 and β13–β17). Six β-strands (β14–β13–β1–β5–β3–β17) form the central β-sheet, flanked on one side by α1–α4 and the N-terminus of α5, and on the other by β15, β16, and α19. Strands β2 and β4, together with helix α20, cap the underside of this central β-sheet. Domain B consists of 14 α-helices (the C-terminus of α5 and α6–α18) and seven β-strands (β6–β12). Five β-strands (β6–β12–β11–β8–β7–β17) form the central core, wrapped by the 14 α-helices and β9–β10.

The two protomers form a head-to-tail dimer, burying ∼4440 Å² of solvent-accessible surface at the interface, with extensive contacts between domain A of one protomer and domain B of the other. NAM binds within a conserved pocket (Fig. 2C–E) formed by residues Phe189, Arg192, Asp215, Ala242, Arg306, and Asp13′, as well as Tyr15. The nicotinamide ring is sandwiched between the side chains of Phe189 and Tyr15′ via parallel π-stacking interactions, while Asp215 forms a hydrogen bond with NAM (Fig. 2D and E).

### Crystal structure of Namat bound to NAD⁺

**Figure 3.**
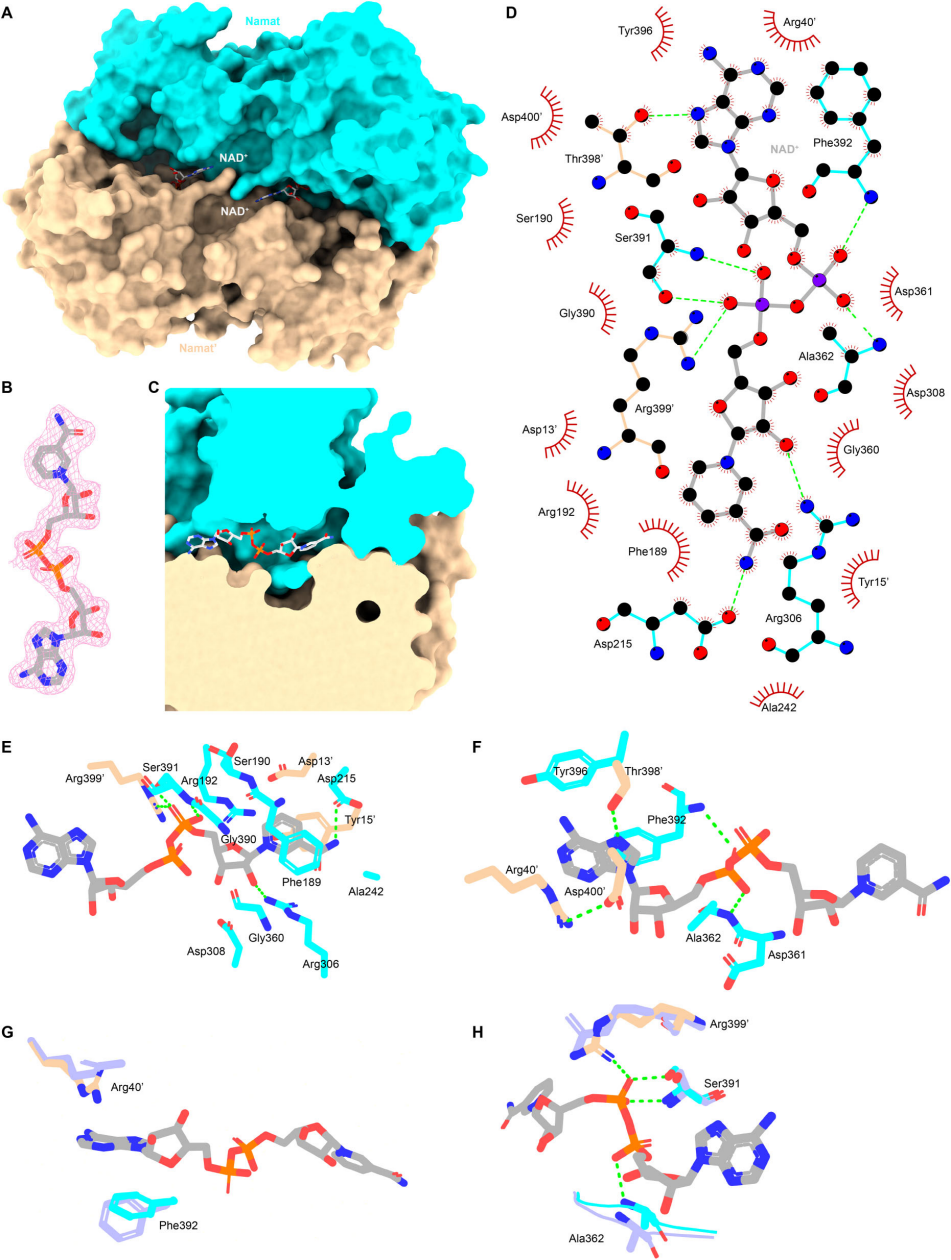
Crystal structure of SpβL1 Namat in complex with NAD^+^. (**A**) Surface representation of Namat dimer in complex with NAD^+^. (**B**) The 2mFo-DFc electron density map (contoured at 1.0 sigma) of NAD^+^ is shown as magenta mesh. (**C**) Slice view of the NAD^+^-binding pocket. 2D LigPlot (**D**) and stereo ribbon diagrams (**E, F**) of key interactions between Namat residues and NAD^+^. Residues involved in hydrophobic interactions are shown as spoked arcs. Hydrogen bonds are indicated by green dashed lines. (**G, H**) Superposition of the structure of Namat-NAD^+^ and Namat-NAM (light blue), revealing conformational shifts in NAD^+^-binding residues.

To further investigate how Namat accommodates its product, we solved the 2.3 Å-resolution crystal structure of Namat in complex with its product NAD⁺ (Fig. 3A, Supplementary Fig. S3A, and Supplementary Table S1), in which the electron density of NAD⁺ was well defined (Fig. 3B). NAD⁺ resides in a deep pocket at the Namat dimer interface (Fig. 3C) and forms extensive interactions with residues Phe189, Ser190, Arg192, Asp215, Ala242, Arg306, Asp308, Gly360, Asp361, Ala362, Gly390, Ser391, Phe392, and Tyr396 of one protomer, and Asp13′, Tyr15′, Arg40′, Thr398′, Arg399′, and Asp400′ of the other (Fig. 3D).

For clarity, we describe these interactions by dividing NAD⁺ into two major components: NMN and AMP. NMN is composed of NAM and one R5P, and AMP consists of adenine attached to a second R5P. The NAM-binding residues are identical to those in the Namat–NAM structure (Fig. 3D and E). The R5P ribose of NMN forms a hydrogen bond with Arg306 and interacts with Asp308 and the backbone of Gly360 (Fig. 3E). The R5P phosphate group of NMN contacts Ser190 and Gly390, donates hydrogen bonds to Ser391, and forms a salt bridge with Arg399’ (Fig. 3E). The AMP moiety lies at the entrance of the binding pocket (Fig. 3A and C). Its phosphate interacts with Asp361 and the backbone of Ala362, while its ribose stacks against Ala362 and Phe392 (Fig. 3F). The adenine base is sandwiched between Phe392, Arg40′, and Asp400′ via π-stacking and is further stabilized by Tyr396 and Thr398′ through hydrogen bonding and π-stacking (Fig. 3F). Notably, Arg40′ and Asp400′ also form a salt bridge (Fig. 3F).

The Namat–NAD⁺ structure shows no major conformational changes compared with the Namat–NAM complex (overall Cα RMSD = 0.160 Å; Supplementary Fig. S3B). Nearly all NAD⁺-binding residues adopt the same conformations as in the Namat–NAM complex, except for Ala362, Ser391, Phe392, Arg40′, and Arg399′, which shift to accommodate NAD⁺ (Fig. 3G and H).

### Namat retains a Nampt-like catalytic core but shifts substrate preference to ADPR–PP

**Figure 4.**
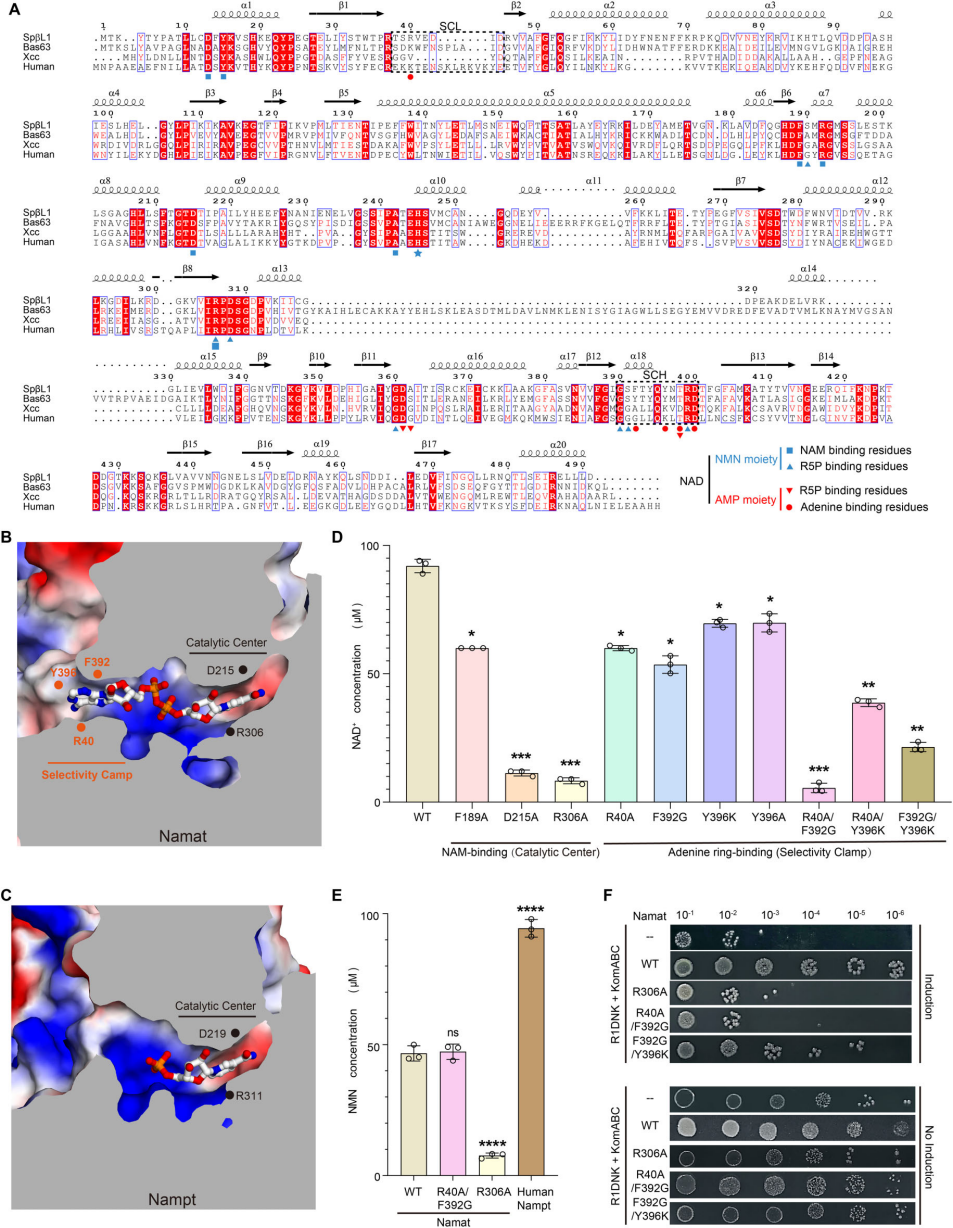
The adenine-recognition clamp governs Namat’s substrate selectivity toward ADPR-PP. (**A**) Sequence alignment of SpβL1 Namat, Bas632 Namat, Xcc Nampt, and human Nampt. Residues contacting NAM and R5P group in NMN moiety are marked by light-blue squares and upward triangles, respectively. And residues contacting R5P and Adenine group in AMP moieties are marked by red downward triangles and circles, respectively. The SCL and SCH are boxed, and the catalytic histidine is indicated by a red star. (**B, C**) Slice view of the substrate-binding pocket of Namat and Nampt (PDB code 2H3D), where blue, red, and white represent positively charged, negatively charged, and neutral areas, respectively. Key residues forming the conserved catalytic center and the Namat specific adenine-dependent ADPR-PP selectivity clamp are indicated by orange and black circles, respectively. (**D**) *In vitro* NAD^+^ generation assays with recombinant Adps, Namat (WT or mutants), ADPR, ATP, and NAM at 30°C; NAD^+^ was quantified using a commercial NAD^+^/NADH assay kit. Data are presented as mean ± s.d (*n* = 3). (**E**) *In vitro* NMN generation assays with recombinant Namat (WT or mutants), PRPP, and NAM at 30°C, with human Nampt was used as a positive control; NMN was quantified using a fluorescence-based method. Data are presented as mean ± s.d (*n* = 3). Statistical significance was determined by one-way analysis of variance followed by Bonferroni multiple comparisons. *P < .05, **P < .01, ***P < .001, and ****P < .0001; n.s, not significant. (**F**) *In vivo* assays in *E. coli* MG1655 co-transformed with plasmids encoding R1DNK, KomABC, and Adps–Namat (WT or mutants). R1DNK expression was induced with L-arabinose and repressed with glucose.

Namat is homologous to Nampt, whose structures have been determined in both human and the bacterium *Xanthomonas campestris pv. campestris* (Xcc) [48, 53–57]. SpβL1 Namat shares 36.3% sequence identity with both human and Xcc Nampt (Fig. 4A). To investigate how Namat discriminates between ADPR-PP and PRPP, we compared its structure with those of human and Xcc Nampt. Structural superposition yielded overall Cα RMSDs of 0.978 Å (versus Xcc Nampt, Supplementary Fig. S4A) and 1.326 Å (versus human Nampt, Supplementary Fig. S4B). Whereas Nampt strictly recognizes PRPP, Namat has switched its specificity to the bulkier ADPR-PP, an analog carrying an additional AMP moiety. The NMN-binding residues, including the catalytic histidine phosphorylated for activation [56], are highly conserved between Namat and Nampt (Fig. 4A). Together with the enzymatic evidence that Namat can utilize both PRPP and ADPR–PP yet strongly prefers ADPR–PP to drive direct NAD⁺ synthesis [33], these features indicate that Namat and Nampt share a common chemical mechanism for NAM attachment to the ribosyl group while differing in their preferred phosphoribosyl donor.

Thus, Namat retains a Nampt-like catalytic core but has shifted its substrate from PRPP to ADPR-PP, implying that the determinants of substrate specificity lie outside the conserved catalytic center, most likely within the AMP–binding portion of the active site.

### An active-site entrance clamp in Namat underlies adenine-dependent ADPR–PP recognition

The most prominent difference between Namat and Nampt lies at the entrance of their substrate-binding pockets (Fig. 4B and C). Namat features a markedly narrower pocket opening that forms a highly specific recognition site for the adenine ring (Fig. 4B). The residues responsible for adenine-ring recognition are poorly conserved (Fig. 4B), indicating that these nonconserved interactions are the principal determinants underlying Namat’s distinct substrate selectivity. We therefore propose that adenine-ring binding residues play a dominant role in shaping Namat’s substrate preference.

Two structural elements together form Namat’s adenine-recognition module, which we define as the ADPR-PP selectivity clamp. The first is the β1–β2 loop, which differs in both sequence and length (Fig. 4A); we refer to it as the SCL. The second comprises α18 and its downstream loop (Fig. 4A), termed the SCH. Acting together, the SCL and SCH shape the narrowed pocket entrance and provide residues that stack against the adenine ring (Fig. 3F).

In Nampt, the regions corresponding to the SCL and SCH (hereafter referred to as the SCL-equivalent loop and SCH-equivalent helix) occupy similar positions, but lack the adenine-binding features of Namat (Fig. 4A). Specifically, the SCL-equivalent loop of Xcc Nampt is shorter (Supplementary Fig. S4A), while the corresponding region in human Nampt is disordered in the crystal structure (Supplementary Fig. S4B). To further assess whether these regions in Nampt can engage the adenine moiety, we examined AlphaFold3 structural models. These models reinforced our conclusion, showing that human and Xcc Nampt lack direct contacts with the NAD⁺ adenine: neither the SCL-equivalent loop nor the SCH-equivalent helix engages the adenine base (Supplementary Fig. S4C and D), consistent with their preference for PRPP over ADPR-PP.

Collectively, these structural analyses indicate that Namat retains the conserved NAM–R5P coupling mechanism of Nampt but has evolved a unique ADPR-PP selectivity clamp that introduces adenine-binding residues and thereby enforces ADPR-PP selectivity. We next examined how the conserved catalytic core and the selectivity clamp contribute to NAD^+^ regeneration.

### The Namat selectivity clamp defines ADPR–PP–driven NAD⁺ synthesis while maintaining NAM–PRPP catalysis *in vitro*

To test our hypothesis that the catalytic core mediates conserved chemistry, whereas the SCL/SCH clamp enforces adenine-dependent substrate selectivity in Namat, we conducted site-directed mutagenesis and *in vitro* NAD⁺ biosynthesis assays.

For the ADPR–PP–dependent reaction, purified recombinant Adps and Namat (WT or mutant) were incubated with ADPR, ATP, and NAM. Consistent with Namat sharing the same catalytic mechanism as Nampt, single-point mutations in catalytic center markedly impaired activity (Fig. 4D). Relative to wild-type Namat, mutants F189A, D215A, and R306A reduced NAD⁺ production by ∼1.5-, 8.1-, and 11.0-fold, respectively. These findings are consistent with the high structural conservation of the catalytic core and the recent characterization of the Namat [33], which showed that substituting the corresponding Arg306 with glycine abolishes NARP1–dependent NAD^+^ reconstitution both *in vitro* and *in vivo*. Together, these independent data sets establish that an intact, conserved catalytic center is essential for Namat activity.

We next asked whether elements outside the conserved core are comparably important for activity. To evaluate the role of the selectivity-clamp residues in substrate recognition, we introduced mutations in the SCL and SCH. Individual mutations in either element (SCL or SCH) modestly reduced NAD⁺ biosynthetic activity (~1.3- to 1.7-fold; Fig. 4D), underscoring that neither element alone dictates specificity. Strikingly, pairwise mutations that simultaneously perturb both clamp, e.g. R40A/F392G, R40A/Y396K, and F392G/Y396K, dampened catalysis by ∼16.6-, 2.4-, and 4.3-fold, respectively. These results demonstrate that the SCL and SCH cooperate as a selectivity clamp to enforce ADPR-PP specificity, with a functional importance comparable to the catalytic core.

We also examined the ancestral Nampt–like activity of Namat. Consistent with previous work [29], *in vitro* assays confirmed that Namat can convert PRPP and NAM into NMN in the absence of ADPR-PP, albeit with approximately half the activity of human Nampt (Fig. 4E). Notably, the double selectivity clamp mutant R40A/F392G, which severely impairs ADPR-PP–dependent NAD⁺ production, retains NMN-producing activity at levels similar to wild-type Namat. By contrast, the catalytic-core mutant R306A markedly disrupts both NAD⁺ and NMN synthesis (Fig. 4D and E). These observations indicate a clear division of labor: the catalytic core is required for both ADPR–PP– and PRPP–dependent reactions, whereas the selectivity clamp is specifically required for the additional ADPR–PP–driven NAD⁺ regeneration pathway and has little impact on the ancestral NAM–PRPP chemistry.

Taken together, these biochemical data show that conserved catalytic residues define the core chemistry shared with Nampt, while the nonconserved SCL/SCH selectivity clamp defines ADPR–PP–driven NAD⁺ synthesis and enforces adenine–dependent substrate choice *in vitro*.

### Both the selectivity clamp and catalytic core of Namat are required to counter NAD⁺-depleting defenses *in vivo*

We next investigated whether the catalytic core and the selectivity clamp are both required for Namat function in the context of NAD^+^-depleting bacterial defenses.

The Kongming (KomABC) system is a recently discovered bacterial defense module [27] that triggers abortive infection by depleting cellular NAD⁺. Upon phage entry, the virally encoded deoxynucleotide kinase (R1DNK), together with KomA and host NDK, converts dAMP to the signaling nucleotide dITP. Then dITP activates the NADase activity of the KomB–KomC complex, cleaving NAD⁺ into NAM and ADPR.

To assess whether Adps–Namat can counteract Kongming-mediated NAD⁺ depletion *in vivo, E. coli* MG1655 cells were co-transformed with pBAD-R1DNK, pCDFDuet-KomABC, and pRSFDuet-Adps-Namat (WT or mutants). The expression of R1DNK in *E. coli* is inducible with L-arabinose and repressible with glucose. In line with our *in vitro* results, cells producing wild-type Namat were fully protected against the KomABC system (Fig. 4F). In contrast, the R306A and R40A/F392G mutants abolished defense (Fig. 4F), whereas the F392G/Y396K variant retained partial protection (Fig. 4E). These results indicate that both the catalytic center and the selectivity clamp are required for Namat’s anti-defense activity *in vivo*.

Together, these results demonstrate that conserved catalytic residues define the core chemistry shared with Nampt, while the unconserved adenine-binding residues of the selectivity clamp impose strict ADPR-PP specificity, enabling Namat to counteract NAD⁺-depleting immune systems.

### Phylogenetic analysis reveals distinct evolutionary features of the Namat selectivity clamp compared with Nampt

**Figure 5.**
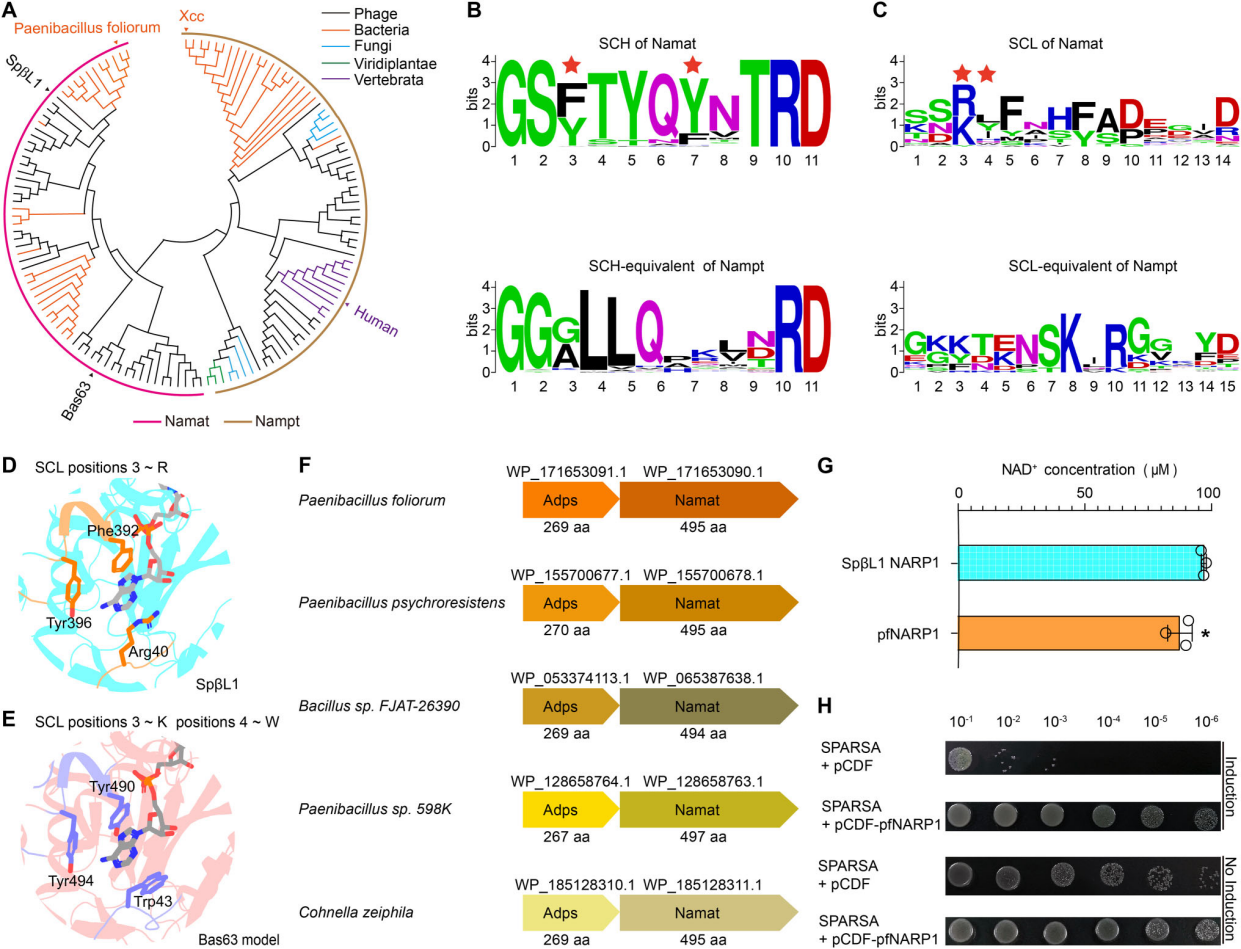
Evolutionary features of the Namat selectivity clamp and identification of NARP1 homologs in bacterial genomes. (**A**) Phylogenetic tree of Namat and Nampt homologs. (**B, C**) Amino acid conservation of the SCH (top) and SCL (bottom) in Namat, compared with their Nampt counterparts. Key adenine-binding residues are marked with red stars. Structural details of adenine-binding interactions in the SCL and SCH of SpβL1 Namat–NAD⁺ crystal structure (**D**) and Bas63 Namat–NAD⁺ AlphaFold3 model (**E**). (**F**) Representative genome organization of bacterial NARP1. (**G**) *In vitro* NAD^+^ biosynthesis by recombinant pfAdps and pfNamat, with SpβL1 NARP1 as the positive control. Data are presented as mean ± s.d (*n* = 3). Statistical significance was determined by two-tailed unpaired Student’s t-test. *P < .05. (**H**) *In vivo* assays in *E. coli* BL21 (AI) co-transformed with pRSFDuet-SPARSA and either pCDFDuet-1 or pCDFDuet-pfNARP1. pfNARP1 expression was induced with IPTG and L-arabinose, and repressed with glucose.

To place these structural features into an evolutionary context, we performed phylogenetic analyses of Namat and Nampt homologs (Fig. 5A and Supplementary Fig. S5). Nampt homologs are broadly distributed across phage, bacteria, and eukaryotes, whereas Namat is restricted to phages and a limited number of bacterial genomes. Amino acid conservation analysis revealed that, compared with the relatively variable SCL and its Nampt counterpart (SCL-equivalent), the SCH and SCH-equivalent exhibit higher sequence conservation (Fig. 5B and C).

Namat has evolved a distinctive consensus sequence absent in Nampt, defined as GSXΦ_1_XΦ_2_XΦ_3_TRD. In this motif, X residues are most often aromatic (Tyr or Phe), whereas Φ residues are typically small polar or polar-neutral amino acids (Fig. 5B), with Φ_1_ predominantly Thr or Ser, and Φ_2_ and Φ_3_ mainly Gln/Asn and Asn/Val, respectively. Importantly, the aromatic residues at positions 3 and 7 directly engage the adenine ring of NAD⁺ (Fig. 5D and E; Supplementary Fig. S6), thereby enforcing ADPR-PP selectivity. This consensus motif thus constitutes a distinctive sequence–structural signature of Namat that differentiates it from Nampt homologs and explains its unique substrate preference.

By contrast, the SCL shows lower sequence conservation and variable length compared with its Nampt counterpart (Fig. 5C). However, structural and AlphaFold3-based analyses revealed that Namat SCL contributes an additional stacking residue for adenine recognition (Fig. 5D and E; Supplementary Fig. S6). The third residue is typically either Arg, Lys, or Tyr. When Arg is present (e.g. SpβL1 Namat), it directly stacks with the adenine base of NAD⁺ (Fig. 5D and Supplementary Fig. S6A); when Lys or Tyr occupies this position, the subsequent fourth residue fulfills the stacking role (e.g. Bas63 Namat, Fig. 5E, Supplementary Fig. S6B–F). Beyond this stacking determinant, the remaining positions within the SCL show higher variability, consistent with the overall lower sequence conservation of this loop compared to the SCH. Thus, the SCL contributes a flexible but critical element of the selectivity clamp, ensuring adenine engagement in cooperation with the more conserved SCH motif.

Together, these phylogenetic and structural analyses highlight a two-layered mechanism for adenine engagement: the relatively conserved SCH provides aromatic stacking residues, while the more variable SCL contributes an auxiliary stacking residue in a position-dependent manner. These complementary features collectively enforce the ADPR-PP selectivity of Namat.

### Identification of NARP1 homologs in bacterial genomes

Our phylogenetic analyses revealed that in most bacterial genomes containing a Namat homolog, the upstream region encodes a gene for an Adps homolog (Fig. 5F), indicating that complete NARP1 modules are also encoded in bacteria. To assess whether bacterial NARP1 can regenerate NAD⁺ like its phage counterpart, we examined the system from *Paenibacillus foliorum*, where pfAdps and pfNamat share 53.6% and 71.9% identity with SpβL1 Adps and Namat, respectively.

*In vitro* enzymatic assays demonstrated that recombinant pfAdps and pfNamat (Supplementary Fig. S7) catalyze the regeneration of NAD^+^ from ADPR and NAM, consistent with the activity observed for phage NARP1 (Fig. 5G). To further examine whether pfNARP1 is functional *in vivo*, we tested its ability to counteract an NAD^+^-depletion immune system. We employed the Sir2-associated short prokaryotic Argonaute immune system (SPARSA) [30, 49], in which the pCDFDuet-1 (ori: CloDF13) construct activates the NADase activity of SPARSA. Co-expression of SPARSA with pCDFDuet-pfNARP1 showed that pfNARP1 effectively neutralized the deleterious effects of SPARSA (Fig. 5H), thereby restoring cellular NAD^+^ levels. These results demonstrate that pfAdps and pfNamat are also capable of regenerating NAD^+^  *in vivo*.

Taken together, our findings suggest that NARP1 is more broadly distributed than previously recognized and may also function in bacteria, although the precise physiological role of bacterial NARP1 requires further investigation.

## Discussion

Our structural and functional analyses define the molecular basis of substrate selectivity in Namat, the central enzyme of the phage NARP1 pathway. Comparison with Nampt reveals that both enzymes share a similar NAM–ribose coupling mechanism, but Namat has evolved distinct selectivity elements: a non-conserved loop (SCL) that contributes an aromatic residue for π-stacking with the adenine ring, and a conserved helix (SCH) that provides two additional aromatic residues absent in Nampt. Together, these elements enforce strict specificity for ADPR-PP rather than PRPP. Mutagenesis and functional assays confirm that the selectivity clamp is as critical as the conserved catalytic center for NAD⁺ biosynthesis and suppression of bacterial NAD⁺-depleting defenses. However, the transplantation of the selectivity clamp into human Nampt did not confer Namat-like activity (Supplementary Fig. S8), indicating that additional structural determinants, likely outside the immediate active site, are required for ADPR-PP recognition.

Beyond structural insights, we identified NARP1-like systems in certain bacterial genomes, consistent with previous genomic surveys reporting bacterial chromosomally encoded NARP1 [58]. Importantly, our work extends these purely bioinformatic and correlative observations by demonstrating that chromosomally encoded bacterial NARP1 indeed exhibits robust ADPR-PP–dependent NAD⁺ biosynthetic activity comparable to their phage counterparts. Thus, we not only corroborate earlier reports that bacteria harbor NARP1-like modules, but also provide direct biochemical evidence that these chromosomal systems are catalytically competent NAD^+^ reconstitution pathways. This represents a previously unrecognized layer of metabolic innovation, raising intriguing questions about their physiological function in bacteria. The predominant chromosomal localization of NARP1 and apyc1, not confined to mobile genetic elements, has been interpreted as evidence that such genes have been stably integrated and domesticated for host-centered functions rather than acting solely as phage-encoded anti-defense factors [58]. In the case of Apyc1, evolutionary and functional analyses have shown that Apyc1 homologs are widespread in nonmobile regions of bacterial genomes and likely originated in bacteria as cNMP-degrading enzymes that regulate housekeeping cyclic nucleotide signaling (for example, cAMP and cGMP), before being co-opted by phages to counteract cNMP-utilizing defense systems [58]. Given the similar predominance of NARP1 and Apyc1 in bacterial chromosomes and their shared association with metabolite-centered immunity and signaling, one plausible scenario is that bacterial NARP1-like systems may have originally evolved as host-encoded NAD⁺ reconstitution modules in the context of bacterial NAD^+^-centered immunity and metabolism, and were later co-opted by phages, most likely through horizontal gene transfer [59] to help counter NAD⁺-depleting defense systems.

In light of this, bacterial NARP1 homologs may function primarily as self-regulatory modules that fine-tune NAD^+^ and ADPR homeostasis to support core redox metabolism, defense and nucleic acid integrity under stress [36, 60]. Recent work in bacteria and their phages has shown that multiple ADP-ribosyltransferases and associated effectors directly modify DNA, RNA and proteins with ADPR, and that dedicated hydrolases reverse these marks, coupling the non-oxidative consumption of NAD^+^ to the control of DNA replication, transcription, RNA processing and stability, and restriction-like or abortive infection responses [23, 24]. In this context, NARP1-mediated NAD^+^ reconstitution may help restore NAD^+^ pools consumed by NADases and ADP-ribosyltransferases during immune activation, buffer intracellular NAD^+^/ADPR fluctuations, and thereby sustain both NAD^+^-dependent dehydrogenases in central redox metabolism and ADPR-based signaling on nucleic acids and proteins, while preventing excessive NAD^+^ depletion that would otherwise bias cells toward irreversible restriction-like or abortive infection outcomes. Future studies combining genetics, quantitative metabolomics, and genome-wide mapping of DNA/RNA ADPR modifications in NARP1-proficient and -deficient bacteria will be required to test these hypotheses and to define the precise contribution of NARP1-like systems to the interface between NAD^+^/ADPR homeostasis and nucleic acid metabolism.

Although our structural analysis of Namat provides critical insight into substrate selectivity, the structure of Adps remains unresolved. Since Adps catalyzes the ATP-dependent phosphorylation of ADPR, its structural characterization will be essential for fully reconstituting the NARP1 pathway at the molecular level. High-resolution cryo-EM or crystallographic studies of Adps, possibly in complex with ADPR or ADPR-PP, would provide a complete mechanistic picture and a foundation for rational engineering of the pathway.

## Supplementary Material

gkaf1492_Supplemental_File

## Data Availability

Atomic coordinates and structure factors for the reported crystal structures have been deposited in the Protein Data bank under accession numbers 9W5X (for Namat-NAM structure) and 9W5W (for Namat-NAD^+^ structure). Other data are available upon reasonable request.
