# Supplementary material for: Structural evolution of the selectivity clamp confers ADPR-PP specificity in Namat, a phage nicotinamide ADP-ribose transferase
Source: Nucleic Acids Res. 2026 Jan 6;54(1):gkaf1492. doi: 10.1093/nar/gkaf1492 (PMC12774641; doi:10.1093/nar/gkaf1492)
Supplement: gkaf1492_Supplemental_File [file gkaf1492_supplemental_file.pdf]

Supplementary material for

Structural Evolution of the Selectivity Clamp Confers ADPR-PP Specificity in Namat, a Phage Nicotinamide ADP-Ribose Transferase

Meimei Lan[1,†], Li Xu[2,†], Yizhen Han[1,†], Tong Cui[1], Zhi Qiao[1], Yanbin Teng[1,*], Na Wang[3,*], Hongyu Bao[1,*]

[1] School of Life Sciences, Anhui Medical University, Hefei, Anhui, 230032, China.

[2] Institute of Bio-Architecture and Bio-Interactions (IBABI), Shenzhen Medical Academy of Research and Translation (SMART), Shenzhen 518107, Guangdong Province, China

[3] School of Life Sciences and Medical Engineering, Anhui University, Hefei 230601, China

* To whom correspondence should be addressed. Email: baohy@ahmu.edu.cn
Correspondence may also be addressed to Na Wang and Yanbin Teng. Email: wangn6@ahu.edu.cn; tengyanbin@ahmu.edu.cn

† Meimei Lan,Li Xu and Yizhen Han contributed equally to this work.

This PDF file includes:

Supplementary Tables S1

Supplementary Figures S1 to S8

Supplementary Table S1. Data collection and structure refinement statistics.

	Namat-NAM	Namat-NAD
**PDB code**	9W5X	9W5W
**Data Collection**	0.9795	0.9786
Wavelength(Å)		
Space group	P1	P1
Cell parameters		
a, b, c (Å)	64.066, 104.74, 119.67	64.04, 104.53, 119.76
α, β, γ (°)	109.31, 101.55, 89.97	109.25, 100.66, 90.36
Resolution (Å)	50.00–2.40	49.21–2.30
	(2.46–2.40)[a]	(2.34–2.30)
R_{merge} (%)	15.4 (47.7)	10.9 (40.2)
$CC_{1/2}$	0.974 (0.760)	0.990 (0.753)
$I/\sigma(I)$	8.3 (2.7)	6.7 (2.9)
Completeness (%)	97.6 (97.2)	90.3 (93.5)
Redundancy	3.4 (3.5)	2.6 (2.7)
**Refinement**		
No. reflections used/free	109433/5318	114969/5612
Resolution (Å)	38.79–2.40	49.21–2.30
R_{work}/R_{free} (%)	22.33/24.00	18.53/22.89
R.m.s.deviations		
Bond lengths (Å)	0.003	0.004
Bond angles (˚)	0.631	0.740
B-factors (Å^2)		
Protein	32.63	28.52
Water	32.32	30.73
Ligand	31.22	24.19
No. atoms		
Protein	15677	15449
Water	610	964
Ligand	36	176
Ramachandran plot		
Favored/allowed/outlier (%)	98.08/1.92/0	97.65/2.35/0

a Values in parentheses are for the highest-resolution shell.

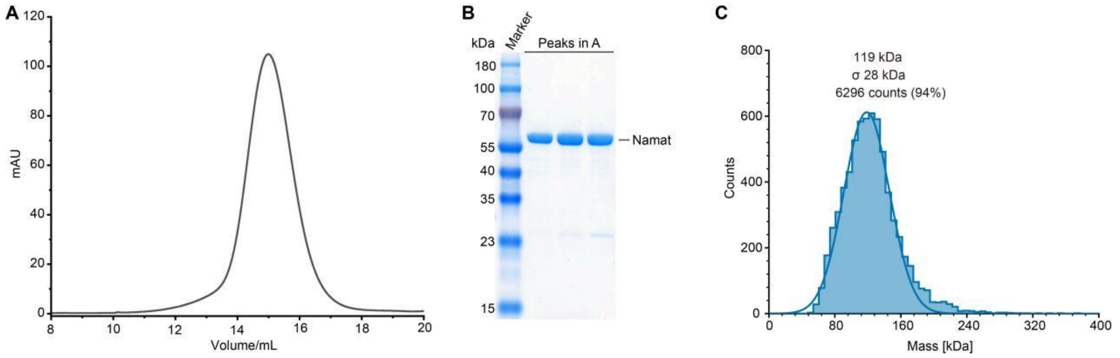

Supplementary Figure S1. SpβL1 Namat is a physical dimer. (**A**) Gel filtration profile of SpβL1 Namat by using Superdex 200 Increase GL columns (Cytiva). (**B**) SDS-PAGE analysis of Namat after gel filtration. (**C**) The molar mass of Namat determined by Refeyn Mass Photometry.

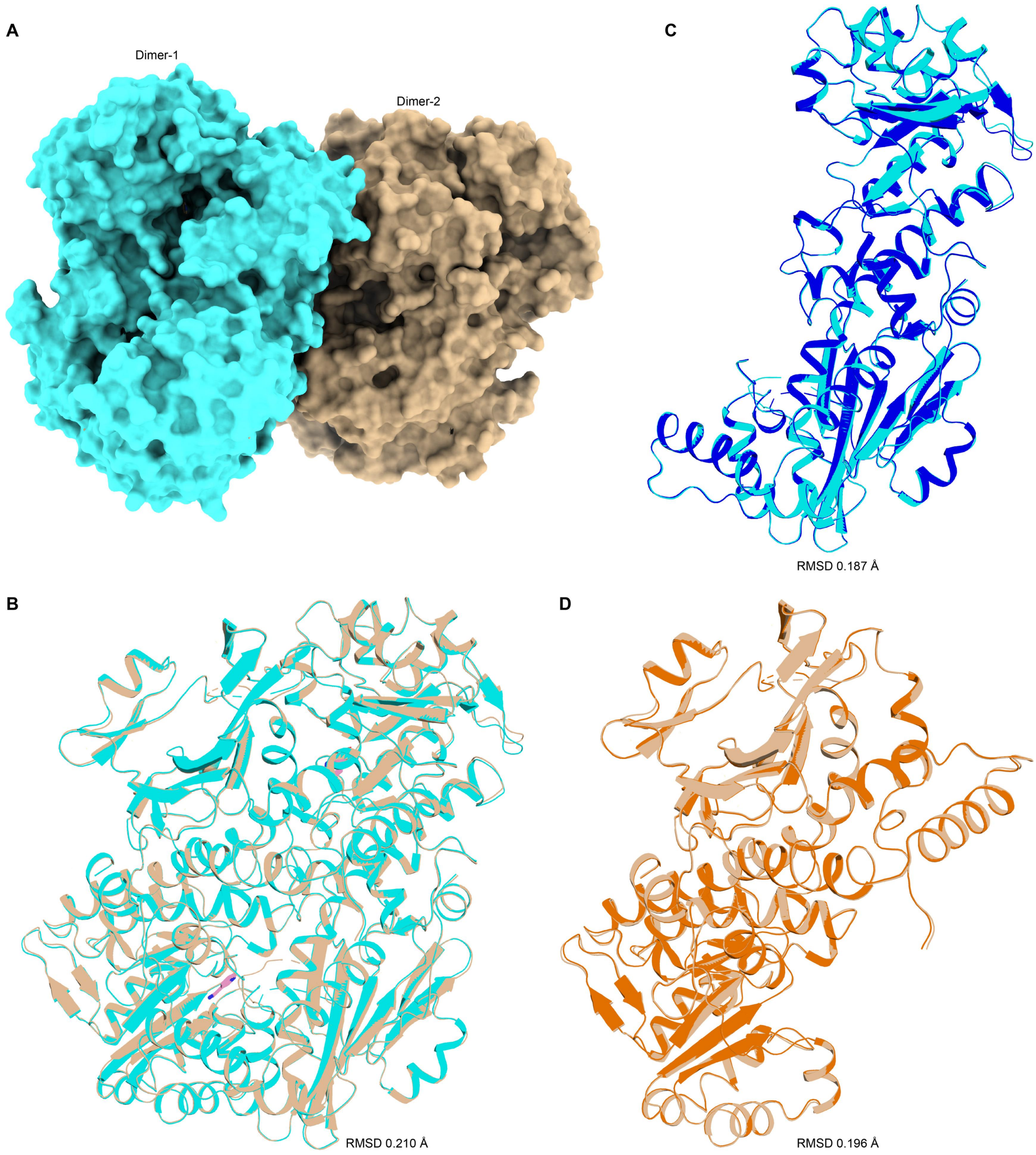

Supplementary Figure S2. Crystal structure of SpβL1 Namat in complex with NAM. (**A**) Surface representation of the asymmetric unit, showing two Namat dimers (four protomers) bound to NAM. (**B**) Superposition of the two crystallographically independent dimers demonstrates near-identity (RMSD = 0.210 Å). (**C, D**) Superposition of the protomers within each dimer, highlighting their high structural congruence(RMSD = 0.187 Å and 0.196 Å).

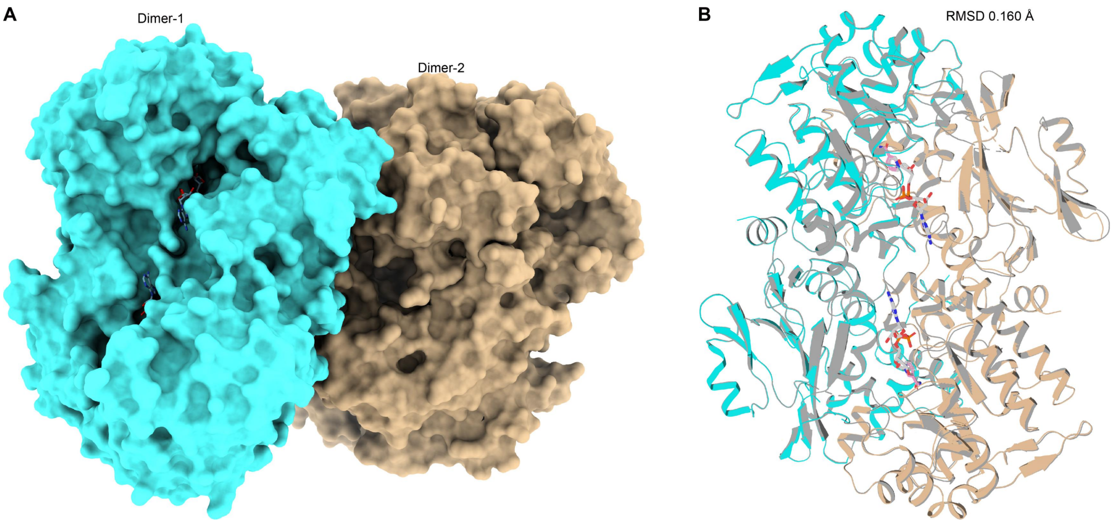

Supplementary Figure S3. Crystal structure of SpβL1 Namat in complex with NAD$^+$. (**A**) Surface representation of the asymmetric unit, showing two Namat dimers (four protomers) bound to NAD$^+$. (**B**) Superposition of Namat-NAM and Namat-NAD$^+$ structures reveals near-identity (RMSD = 0.160 Å).

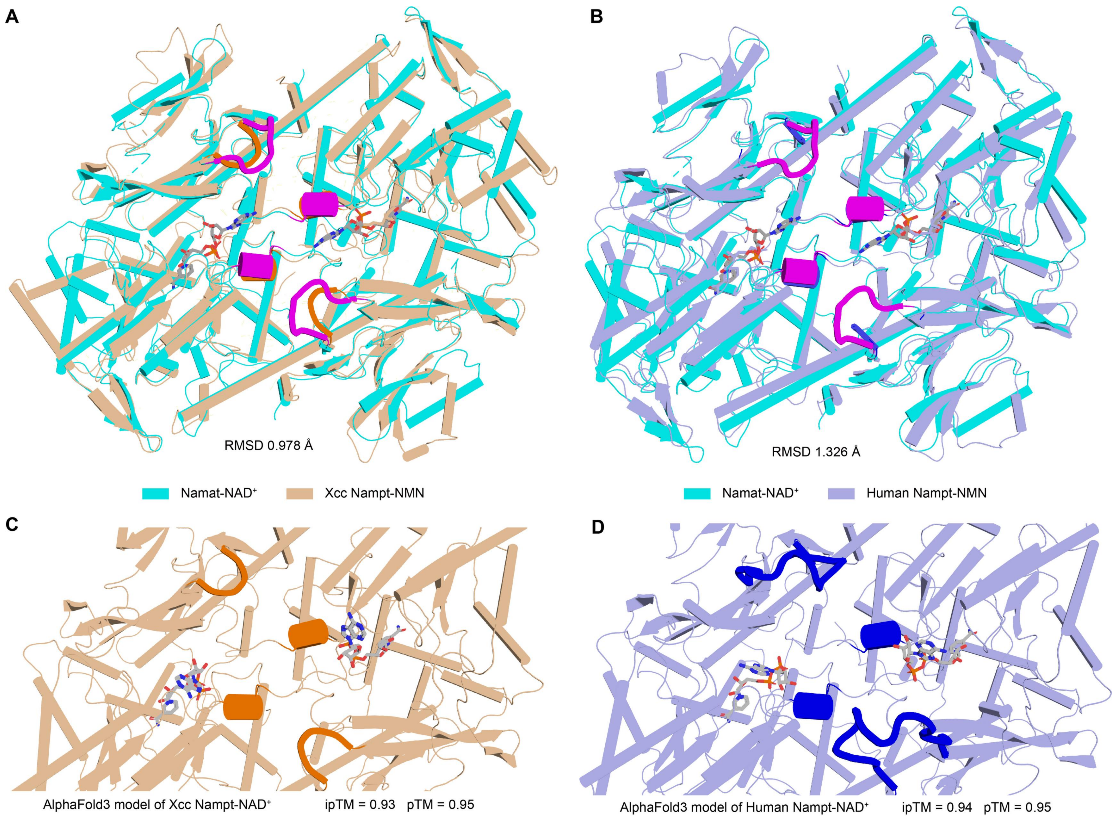

Supplementary Figure S4. Structural comparison of Namat and Nampt. (**A, B**) Superposition of Namat-NAD$^+$ (cyan, with SCL/SCH in magenta) with Xcc Nampt-NMN (PDB code 7YQQ, wheat, SCL-equivalent loop/SCH-equivalent helix in orange) and human Nampt-NMN (PDB code 2H3D, light blue, equivalents in blue). (**C, D**) AlphaFold3 models of Xcc and human Nampt in complex with NAD$^+$.

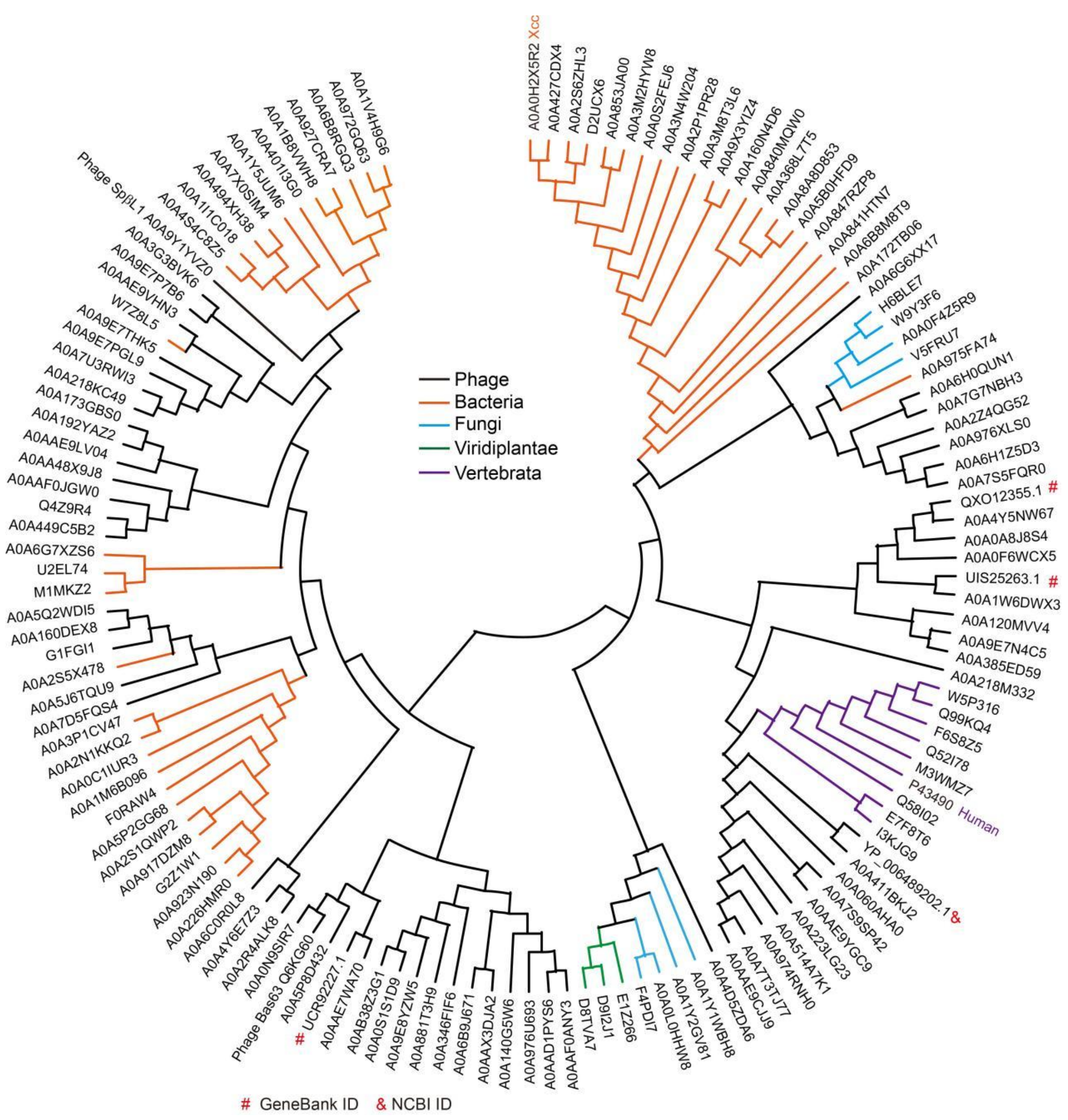

Supplementary Figure S5. Phylogenetic tree of Namat and Nampt homologs. UniProt accession numbers are shown; GenBank and NCBI IDs are marked with '#' and '&,' respectively.

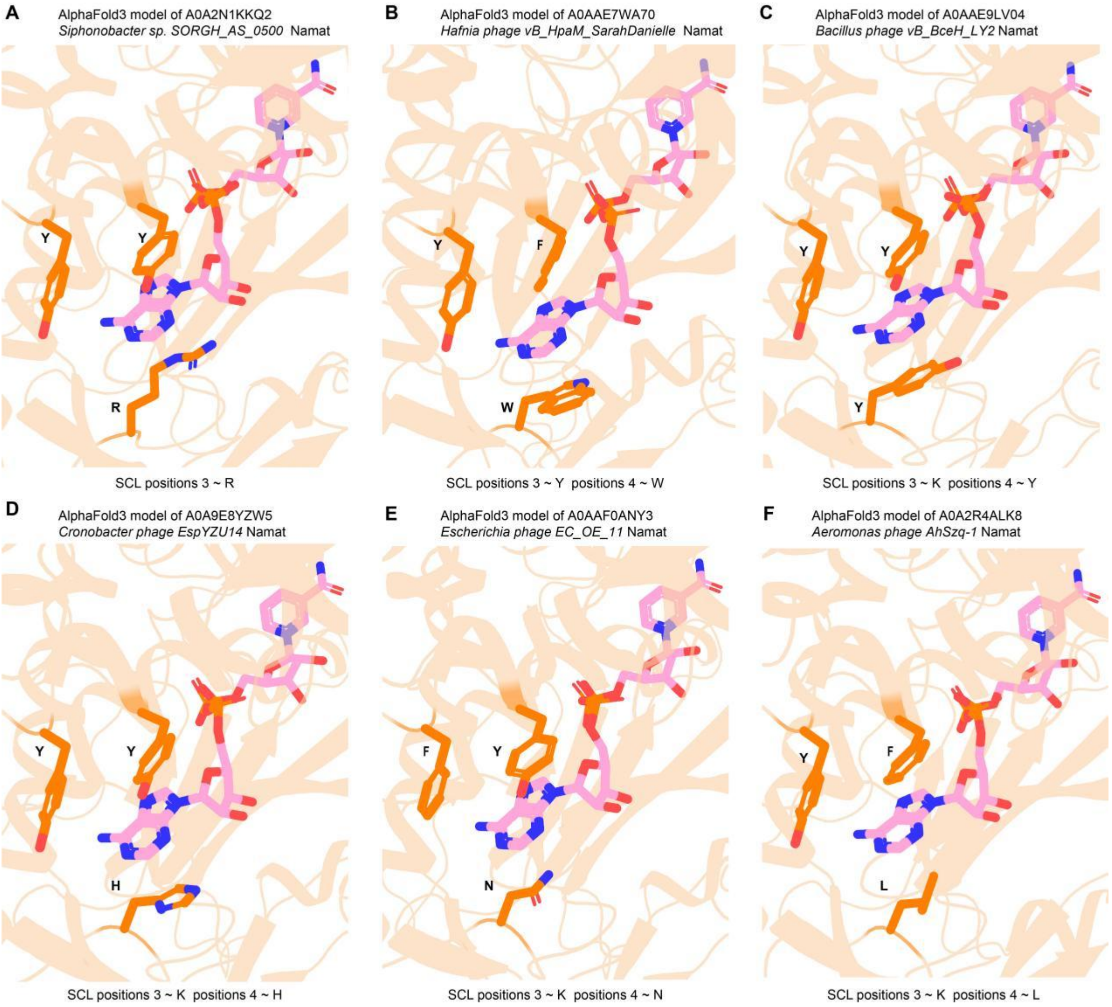

Supplementary Figure S6. AlphaFold3 models revealing evolutionary features of the Namat selectivity clamp. The SCH motif in Namat (top) most frequently contains aromatic residues (Tyr or Phe) at positions 3 and 7, which directly engage the adenine ring of NAD^+. (**A**) In the SCL (bottom), when the third residue is Arg, it directly stacks with the adenine base. (**B-F**) When Lys or Tyr occupies this position, the stacking interaction is instead mediated by the subsequent fourth residue.

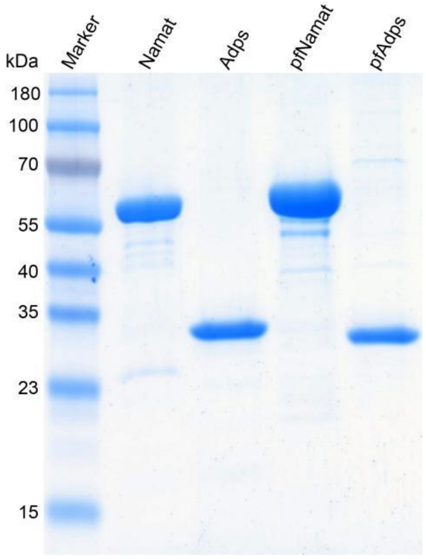

Supplementary Figure S7. SDS-PAGE analysis of purified Namat and Adps. SDS-PAGE analysis of recombinant Namat and Adps from phage SpβL1 and *Paenibacillus foliorum* after size-exclusion chromatography purification. These proteins were used for the in vitro NAD^+ biosynthesis shown in Fig. 5G.

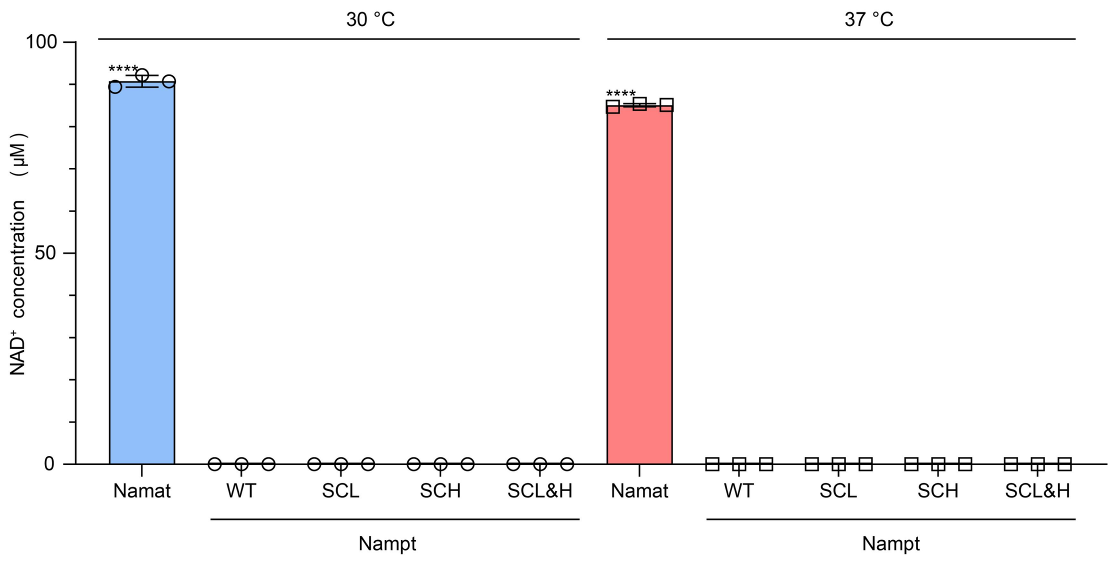

Supplementary Figure S8. In vitro NAD$^+$ biosynthetic assay of Nampt and Namat-like mutants. Assays were performed by co-incubating purified recombinant Adps, human Nampt (WT or Namat-like mutants), ADPR, ATP, and NAM at 30 °C or 37 °C. NAD$^+$ production was quantified using a commercial NAD$^+$/NADH assay kit. SpβL1 Namat served as the positive control. Mutants include: SCL, substitution of the human Nampt SCL-equivalent loop with the SpβL1 Namat SCL; SCH, substitution of residues in the SCH-equivalent helix (G385F/K389Y) with those from SpβL1 Namat SCH; SCL&H, combined SCL and SCH substitutions. Data are presented as mean ± s.d (n = 3).
